# Implementation of enhanced recovery protocols in patients undergoing cytoreductive surgery and hyperthermic intraperitoneal chemotherapy for metastatic ovarian cancer following neoadjuvant chemotherapy. A feasibility study

**DOI:** 10.1016/j.gore.2024.101536

**Published:** 2024-10-28

**Authors:** Anastasios Pandraklakis, Chrysoula Liakou, MariaClelia La Russa, Rocio Ochoa-Ferraro, Adam Stearns, Nikolaos Burbos

**Affiliations:** a Department of Gynaecology and Gynaecological Oncology, Norfolk and Norwich University Hospital, Norwich NR4 7UY, UK; b Department of Anesthesia, Norfolk and Norwich University Hospital, Norwich NR4 7UY, UK; c Department of Surgery, Norfolk and Norwich University Hospital, Norwich NR4 7UY, UK

## Abstract

**Objective:**

The aim of this study is to evaluate the implementation of the elements of enhanced recovery (ERAS) protocols in patients undergoing cytoreductive surgery and hyperthermic intraperitoneal chemotherapy (HIPEC) for metastatic ovarian cancer. ERAS protocols have shown improvement in the perioperative outcomes of patients who underwent cytoreductive surgery for metastatic ovarian cancer by reducing the length of stay as well as the postoperative complications and by improving patients’ postoperative experience**.**

**Methods:**

This is a feasibility study involving retrospective analysis from (31) patients who underwent cytoreductive surgery and HIPEC versus (35) a control group that underwent cytoreductive surgery only, prior to the introduction of the HIPEC programme for metastatic ovarian cancer. All patients had undergone neoadjuvant chemotherapy prior to surgery. We compared the compliance for each element of the ERAS protocol between the two study groups.

**Results:**

We analyzed data from 66 patients, 31 in HIPEC group and 35 in the control goup. We found no significant difference in the patients’ characteristics between the two groups and there were no differences in the implementation of 8 elements of the ERAS protocols (100 % for both groups). The use of nasogastric tube was more frequently observed in patients undergoing surgery and HIPEC compared to those undergoing surgery alone (42 % vs 0 %, respectively; p < 0.001). The number of patients who were mobilized on the first postoperative day was higher in the group undergoing surgery and HIPEC (87.1 % vs 57.1 %, respectively; p = 0.007), however there was no significant difference in the percentage of patients that had early removal of the urinary catheter (p = 0.12), nor in the percentage of patients that received early feeding (p = 0.18). Finally, there were no statistically significant differences in the complication rates, the length of hospital stay and the re-admission rates between the two groups.

**Conclusion:**

Enhanced recovery protocols can be implemented safely in patients undergoing cytoreductive surgery and HIPEC for ovarian cancer.

## Highlights

• ERAS protocols can be safely implemented in patients undergoing IDS and HIPEC for metastatic ovarian cancer.
• High compliance rates can be achieved withouot increasing the complication rates from the additional HIPEC treatment.
• ERAS protocols can be safely considers as standard perioperative care in these patients.

## 1 Introduction

The enhanced recovery after surgery (ERAS) protocols have been introduced as evidence-based, standardized perioperative holistic and multidisciplinary tools in order to minimize the perioperative inflammatory response caused by major surgery, thus accelerating the postoperative recovery (Ljungqvist et al., Mar. 2017). The implementation of these protocols has shown a reduction of 32% in the postoperative complication rates and a reduction of 1.6 days in the length of stay. (Bisch, 2021) These protocols were initially introduced by colorectal surgeons in the 1990′s (Kehlet, 1997) and they have been proposed in gynaecological oncology since 2016 (Nelson, 2016) with a recent update in 2023. (Nelson et al., 2023).

Cytoreductive surgery and intravenous carboplatin-based chemotherapy are the cornerstone of treatment for patients with metastatic ovarian cancer (Ledermann, 2024). The use of hyperthermic intraperitoneal chemotherapy (HIPEC) can be considered at the time of cytoreductive surgery after neoadjuvant chemotherapy (Ledermann, 2024). However, the surgical complexity of surgery combined with additional HIPEC treatment increases dramatically the patients’ biological response to surgical stress (Webb et al., 2013) and therefore the risk of postoperative complications. (“Hyperthermic Intraperitoneal Chemotherapy in Ovarian Cancer, 2018).

Although ERAS guidelines for the management of cytoreduction with or without HIPEC were published in 2020 by the ERAS Society (Hübner, 2020, Hübner, 2020), there is a disappointingly poor adherence on ERAS protocols in this field and there is a significant variation in the perioperative management of these cases amongst different centres. (Cooksley and Haji-Michael, 2011, Mogal, et al., 2016, Yang, 2023) These variations include early feeding and mobilization versus prolonged admissions to the intensive care units and immobility and the use of a nasogastric tube versus gastrostomy or total parenteral nutrition in order to maintain adequate nutrition levels for these cases (van Kooten, 2021).The above variations in the use of ERAS protocols are likely to be related to the complexity of surgical treatments and clinician’s concerns about the safety of implementation.

The primary aim of this study is to assess the feasibility and the safety of the use of the ERAS protocols in patients who underwent cytoreduction and HIPEC for advanced stage, high-grade serous epithelial ovarian cancer using the closed HIPEC technique. The compliance rates to the ERAS protocol and the impact on the length of stay, the duration of admission in the intensive care unit, the postoperative complications, the readmission rates and the 30-day mortality, were analyzed.

## 2 Materials and methods

A. Study participants and design

This is a feasibility study, performed at a referral gynaecological oncology center in the United Kingdom. We included 31 consecutive patients that underwent interval debulking surgery and HIPEC at Norfolk and Norwich University Hospital over a 30-month period, and 35 consecutive patients (control group) that underwent interval debulking prior to the establishment of the HIPEC programme in the centre. The data presented were collected as part of the clinical governance requirements for the implementation of HIPEC protocols for the treatment of patients with metastatic ovarian cancer. The ERAS protocol has been implemented in our hospital since 2016.

Patients younger than 80 years with no evidence of chronic renal impairment, that underwent cytoreductive surgery for stage IIIC and IV epithelial ovarian cancer after neoadjuvant chemotherapy, received HIPEC if tumour resection to less than 2.5 mm was achieved. The HIPEC procedure was performed using the ‘closed technique’. Cisplatin 100mg per square meter (50% of the dose at 0 min, 25% at 30 min and 25% at 60min) was administered using Performer HT perfusion machine (RanD-Biotech) and intra-abdominal temperature was maintained at 41°C during the HIPEC treatment. Sodium thiosulphate was given during and after the procedure to prevent nephrotoxicity. (“Hyperthermic Intraperitoneal Chemotherapy in Ovarian Cancer,” N. Engl. J. Med., vol. 378, no. 14, 2018, Liakou, et al., 2023) Patients older than 80 years and those with a history of chronic renal impairment were not offered HIPEC at the completion of cytoreductive surgery. The control group consisted of 35 consecutive patients that underwent cytoreductive surgery following neoadjuvant chemotherapy for stage IIIC and IV ovarian cancer in the period immediately prior to introduction of HIPEC in our centre.

We evaluated compliance to ERAS protocols in gynaecological oncology that comprised of 16 elements, describing recommendation of care in the preoperative, intraoperative and postoperative periods. (“Hyperthermic Intraperitoneal Chemotherapy in Ovarian Cancer,” N. Engl. J. Med., vol. 378, no. 14, 2018, Hübner, 2020, Nelson, 2019). Preoperative elements consisted of preadmission counselling and optimization, avoidance of bowel preparation, oral carbohydrate loading, avoidance of long acting sedatives, and thromboprophylaxis administration. Intraoperative elements included the use of antibiotic prophylaxis, agents for the prevention of nausea and vomiting, multimodal analgesia including thoracic epidural analgesia, avoidance of nasogastric tube, goal directed fluid therapy, and maintenance of normothermia. Postoperative elements contained the early removal of the urinary catheter, administration of laxatives, early feeding, early mobilization and a 30-day follow up (Table 1).

**Table 1:** ERAS Elements.

Protocol element	Specific	IDS (%)	IDS + HIPEC (%)	*p*
Preadmission counselling and optimisation	Information, education, nutritional support, recommendation to stop smoking, Hb optimization	100	100	1
No bowel preparation	Rectal enema given the night before the surgery	100	100	1
Oral carbohydrate loading	No fasting, carbohydrate loading liquid 3 h before surgery.	100	100	1
Avoid long acting sedatives	Avoidance of long-acting sedatives starting from day before surgery	100	100	1
Thrombosis prophylaxis	Prophylactic LMWH 12 h prior to the operation and 6 h postoperatively, continuing for a 28-day duration	97.1	100	0.86
Antibiotic Prophylaxis	Intravenous antibiotics 60mins before the start of the operation	100	100	1
PONV prophylaxis	Multimodal approach: Dexamethasone in induction and ondansetron after the end of the operation	100	100	1
Goal-directed fluid therapy	Cardiac output monitor was used to monitor fluid vasopressor requirements (invasive)	100	100	1
Normothermia maintenance	Upper body forced-air heating cover and warmed intravenous fluids were used routinely to keep body temperature > 36 °C. During HIPEC cold intravenous fluids and placement of ice packs in the axillae of the patient may be required to normalize the temperature.	100	100	1
Analgesia	Multimodal analgesia including thoracic epidural analgesia	94.2	100	0.34
NGT	Avoidance of NGT postoperatively	100	58	<0.001
Urinary catheter removal	Urinary catheter removal on POD 3	45.7	64.5	0.12
Laxatives	Docusate sodium starting on POD 1	100	100	1
Early feeding	Oral fluids the day of the operation and liquid or light diet (>300 kcal) the day after the operation	82.9	93.5	0.18
Early Mobilization	Active mobilization on POD 1, with full mobilization on POD 3	57.1	87.1	0.007
30-day follow up	Outpatient follow up	100	100	1
Total (mean)		92.3	93.9	

LMWH – Low molecular weight heparin

PONV – postoperative nausea and vomiting.

NGT – nasogastric tube.

POD – Postoperative day.

During preoperative outpatient assessment, the patients were provided with information regarding the extent of the operation as well as their provisional perioperative care, and probiotic supplements were added to their diet. Patients with hemoglobin (Hb) levels below 100g/dl were receiving intravenous iron infusion or transfusion if Hb was below 80g/dl. On the day before surgery, the patients were admitted on the ward and low molecular weight heparin was given 12 h before the start of the operation. A rectal enema was administered the night before the procedure in addition to intravenous fluids (3 L of isotonic fluids over 12 h) in order to maintain high hydration levels. The patients also received a loading dose of carbohydrates 3 h before introduction to anaesthesia.

In the anesthetic room, intravenous antibiotics were given 60 min before skin incision and the ERAS anesthetic protocol was followed (total intravenous anesthesia, thoracic epidural analgesia, multimodal analgesia, goal-directed fluid therapy, multimodal antiemetic medication, active warming) to all the patients. The group of patients underwent HIPEC during the administration of the heated chemotherapy received cold intravenous fluids and placement of ice packs in the axillae of the patients to normalize their temperature. All of the patients were extubated in the operating theatre after the end of the procedure and were transferred to the intensive care unit. Nasogastric tubes were removed before extubation, and two abdominal drains were maintained and later removed on the ward on postoperative day 2, after ensuring that the patients were fully mobile. Epidural analgesia was used in combination with the multimodal analgesic protocol and the epidural catheters and urinary catheters were removed on postoperative day 3. Oral nutrition with fluids started immediately after the operation and solid light diet, laxatives and active mobilization (chest physiotherapy, mobilization with physiotherapists and specialist nurses) started on postoperative day 1. Discharge was based on criteria which consisted of sufficient mobility for self-care, pain control with oral analgesia, diet with solid food, passing of flatus and no complications that required hospitalization. All patients were followed up within 30 days postoperatively. The compliance score was calculated as the sum of individual compliance for each element divided by the total number of elements (16-elements).

We analysed patient demographics, clinical characteristics, ERAS compliance, and outcomes of interest with descriptive statistics. Surgical complexity was stratified in accordance with the Aletti scoring system into low (≤3), medium (4–7) and high (≥ 8) groups. (Aletti, 2007, Aletti et al., 2007) We compared clinical and demographic characteristics between the groups using the chi-squared test or z-score for categorical variables and the Student’s *t* test for continuous variables. P values less than 0.05 were considered significant. No adjustments were made for multiple comparisons. All analyses were completed using Wizard (Version 1.9.49). The structure of the study was based on the recommendations of the Strengthening the Reporting of Observational Studies in Epidemiology (STROBE) statement (von Elm et al., 2014) and on the ERAS RECOvER Checklist recommendations by the ERAS® Society (Elias, 2019).

## 3 Results

A. Clinical Characteristics

A total of 31 patients underwent cytoreductive surgery and HIPEC and 35 patients underwent surgery only. All patients were diagnosed with high-grade serous carcinoma. There was no significant difference in the age of the patients (p=0.07), body mass index (p=0.96) and American Society of Anesthesiologist physical status (ASA) (p=0.42) between the two groups (Table 2). The majority of patients in both groups had stage IIIC disease. There was no difference in the surgical complexity score between the two groups (p=0.29). 8 (25.8%) patients in the HIPEC group and 9 (25.7%) patients in the control group underwent large bowel resection. Amongst the patients that underwent bowel resection, the rate of rectosigmoid anastomosis was significantly higher in the HIPEC group compared to the control (75% vs 22.2%, p=0.021). The mean duration of the surgical procedure was significantly increased for the HIPEC group compared to the control group (537 vs 334 min, p<0.001).

B. ERAS protocol compliance

**Table 2:** Clinical characteristics.

	IDS (n = 35)	IDS + HIPEC (n = 31)	*p value*
Age, mean (range)	67.2 (42–79)	63.7 (52–74)	0.07
BMI (kg/m^2^), mean (SD)	26.4 (4.93)	26.3 (4.68)	0.96
ASA score	0.42		
1	0	0	
2	10	7	0.57
3	25	23	0.80
4	0	1	0.28

Tumor histologic type			
N (%)			
High grade serous	35 (100 %)	31 (100 %)	1

*FIGO staging*	0.59		
IIIC	23 (65.7 %)	17 (54.8 %)	
IVA	3 (8.6 %)	5 (16.1 %)	
IVB	9 (25.7 %)	9 (29.1 %)	

Surgical Complexity Score (*Aletti score*)			0.246
Low (≤3)	13	7	0.20
Intermediate (4–7)	16	18	0.55
High (≥8)	6	6	0.26
Operating time, min, mean (SD)	334 (141)	537 (147)	<0.001

IDS – Interval Debulking Surgery.

HIPEC − Hyperthermic intraperitoneal chemotherapy.

BMI – Body mass index.

ASA − American Society of Anesthesiologists Physical Status Classification System.

SD – Standard deviation

Total mean compliance rates for the ERAS protocols were similar between the two groups (93.9% HIPEC group vs 92.3% control group) (Table 2). The use of nasogastric tubes postoperatively was significantly higher in the group that underwent HIPEC compared to the control group (42% vs 0%, p<0.001) Furthermore, significantly improved mobilization rates on the first postoperative day were observed in the HIPEC group (87.1% vs 57.1%, p=0.005). Non statistically significant differences were observed regarding the percentage of patients that had early feeding (p=0.18), on the rate of preoperative thrombosis prophylaxis p=0.87), on the use of multimodal analgesia protocol (p=0.34) and on the percentage of patients that had urinary catheter removal on postoperative day 3 (p=0.12). Compliance rates of the remaining 8 ERAS elements were 100% in both groups. All elements of the ERAS protocol were applied to 19 (61.2%) patients in the HIPEC group and 15 (42.8%) in the control group.

C. Postoperative Outcomes

There was no significant difference in the length of stay in the intensive care unit (2 days, control group vs 3 days, HIPEC group, p=0.71), and the duration of hospital stay (7 days control group versus 8 days HIPEC group, p=0.52) (Table 3). In addition, no differences were detected in grade III/IV postoperative complication rates between the two groups (3.2% HIPEC group vs 5.7% control group, p=0.63). More specifically, one patient in the HIPEC group developed acute kidney injury postoperatively that required dialysis. This led to changes in the treatment protocols and use of sodium thiosulphate was introduced for all patients receiving HIPEC. Two patients in the control group developed pleural effusion that required drainage under local anesthesia. The rate of hospital readmissions was similar in both groups. There were no deaths within 30-days following the surgery.

**Table 3:** Postoperative outcomes.

	IDS (n = 35)	IDS + HIPEC (n = 31)	*p value*
LOS, median (IQR) days	8 (6–9)	7 (6–9)	0.52
HDU, median (IQR) days	2 (1–3)	3 (1–4)	0.71

30 −day complications (Clavien Dindo System)			
Any	14 (40 %)	16 (51.6 %)	0.34
I-II	12 (34.2 %)	15 (48.3 %)	0.35
III-IV	2 (5.7 %)	1 (3.2 %)	0.63

Re-operation	0	0	1
Hospital re-admission	2	2	0.90
Mortality	0	0	1

LOS – Length of hospital stay (days).

HDU – Length of stay in high dependency unit (days).

Clavien–Dindo *peri*-operative complication occurring within 30 days of surgery.

## 4 Discussion

In this study, we evaluated the feasibility and safety of the implementation of ERAS protocols in patients who underwent HIPEC following interval debulking for metastatic epithelial high-grade ovarian cancer. Our results revealed similar mean compliance rates on a 16-element ERAS protocol for both groups. However, when each element was compared separately, we found a significant increased use of nasogastric tubes in patients who underwent HIPEC (42% vs 0%, p<0.001). It is likely this result reflects our initial concerns about the effect of the intraperitoneal chemotherapy and hyperthermia on bowel function postoperatively. The use of nasogastric tubes was abandoned after the first 10 cases that underwent cytoreductive surgery and HIPEC. Also, we found that significantly more patients in the HIPEC group were mobilised in the early postoperative period compared to control group (87.1% vs 57.1%, p=0.007). This is due to the additional daily mobilization program provided by the specialist gynecological oncology nurses. We found no significant difference in the rates of postoperative outcomes between the two groups.

Regarding the hypothesis that HIPEC can be an additional comorbidity factor on the already high complexity management of ovarian cancer patients, the use of ERAS protocols can potentially help control this excessive response to intraoperative stress and therefore minimise morbidity. (Ljungqvist et al., Mar. 2017) There are several other factors affecting morbidity rates of these patients including age, surgical complexity, and comorbidities. In our study cisplatin toxicity was effectively avoided with the use of sodium thiosulphate. Zhao et al. in their *meta*-analysis (Zhao, et al., 2020) and van Driel et al. in an RCT found that HIPEC treatment did not cause additional morbidity to these petients (“Hyperthermic Intraperitoneal Chemotherapy in Ovarian Cancer, 2018). Antonio et al. showed that there was no statistically significant increase in complications and morbidity in patients treated with additional HIPEC (58.3% vs 45.7%, p>0.05 and 2.8% vs 2.9%, p>0.05) (Antonio, 2022) and although our results were similar to the current literature, revealing no additional morbidity after the implementation of HIPEC, these treatments should only be applied by experienced multidisciplinary teams.

Considering the increased risk of hemodynamic instability, electrolyte imbalances and postoperative complications, patients should be thoroughly followed up after surgery. There is however a variation of opinions regarding the perioperative management of these complex cases. Cooksley and Haji-Michael in their retrospective published audit, suggest a checklist for HIPEC patients including the use of epidural analgesia, early extubation, intensive care unit admission, total parenteral nutrition and fluid balance monitoring(Cooksley and Haji-Michael, 2011). However, Mogal et al. suggested that routine admission to the intensive care unit is not required for all cases and that it is recommended only in selected patients with multiple risk factors (age, multiple organ resections, frailty and nutritional status, blood loss). (Mogal, et al., 2016) In this study, patients who were transferred straight to the ward had less grade I/II complications and similar grade III/VI complications. Yang et al. showed that standardized fluid management protocol, using total parenteral nutrition postoperatively, can reduce cardiovascular complications. (Yang, 2023) Swain et al. in their study, suggested a routine use of total parenteral nutrition to all complex HIPEC cases in order to maintain the nutritional effect while outweighing the disadvantages of this method (infection, refeeding syndrome, thrombosis). (Lu, 2020) Furthermore, van Kooten et al. showed a statistical improvement in the length of stay (15 days vs 18,5 days, p<0.001) in colorectal patients managed with nasogastric tubes after HIPEC comparing to a group of patients with percutaneous gastrostomy for prophylactic gastric decompression. In our study, patients were actively mobilized the day after surgery and early oral intake began on the day after the procedure. The complication rates in the group of patients receiving HIPEC treatment were similar to the results of the published RCTs. (“Hyperthermic Intraperitoneal Chemotherapy in Ovarian Cancer,” N. Engl. J. Med., vol. 378, no. 14, 2018, Antonio, 2022, Lim, 2022).

ERAS guidelines on the management of patients undergoing cytoreductive surgery with or without HIPEC were mainly based on data from studies in patients with colorectal cancer. (Hübner, 2020, Hübner, 2020) Lu et al. in their study, showed that, after the application of ERAS protocols in patients who underwent cytoreduction followed by HIPEC, length of stay was decreased by 3 days without any significant difference in postoperative complication and readmission rates. (Lu, 2020) A recent *meta*-analysis on ERAS protocols in patients who underwent cytoreduction with or without HIPEC for peritoneal cancer, with data mostly from colorectal cancer patients, also confirmed the safety of the ERAS programme and showed significant improvement in the length of stay and complication rates when high compliance was achieved. (Robella, 2023) Although our study is based on a small sample size, it demonstrated comparable rates of grade III/IV complications, length of stay and compliance rates to the ERAS protocol guidelines in patients undergoing cytoreductive surgery with or without HIPEC. These findings suggest that ERAS elements can be safely implemented in patients undergoing cytoreductive surgery and HIPEC with cisplatin for metastatic ovarian cancer. In addition, we demonstrated a high compliance rate with ERAS elements, confirming that ERAS protocols can be successfully implemented in centres with experienced teams.

## 5 Strengths and Weaknesses

The strengths of our study consist of the fact that all the patients were treated in a tertiary gynaecological cancer centre, by gynaecological oncology surgeons experienced in cytoreductive surgery for metastatic ovarian cancer. Similarly, all the anesthetic procedures were performed by anesthetists experienced in goal-directed fluid management and in perioperative and postoperative ERAS protocols. The main limitations of the study include the small number of patients presented and its single-centre design. In addition, although there are several ERAS protocols available, for practical purposes we decided to study only the implementation of the protocol containing 16 elements.

## 6 Implications for further research

Further studies are required to evaluate the role of ERAS protocols in patients that undergo bowel resection and anastomosis as part of the cytoreductive surgery and HIPEC for metastatic ovarian cancer.

## 7 Conclusions

We present the results of a study assessing the feasibility and safety of the implementation of ERAS protocols in patients who underwent interval cytoreductive surgery and HIPEC for metastatic ovarian cancer. Our study showed that the elements of the ERAS protocol studied can be safely implemented in the care of these patients. In addition, achieving high compliance rates in the ERAS protocol did not affect negatively the duration of hospital stay or the risk of postoperative complications. There is, however, still a need for further studies to confirm our findings and to provide stronger evidence on the use of ERAS protocols in patients undergoing cytoreductive surgery with HIPEC.

## CRediT authorship contribution statement

**Anastasios Pandraklakis:** Writing – review & editing, Writing – original draft, Methodology, Investigation, Formal analysis, Data curation, Conceptualization. **Chrysoula Liakou:** Writing – review & editing, Data curation. **MariaClelia La Russa:** Writing – review & editing, Data curation. **Rocio Ochoa-Ferraro:** Writing – review & editing, Investigation. **Adam Stearns:** Writing – review & editing, Investigation. **Nikolaos Burbos:** Writing – review & editing, Project administration, Conceptualization.

## Declaration of competing interest

The authors declare that they have no known competing financial interests or personal relationships that could have appeared to influence the work reported in this paper.

## References

[b0005] “Hyperthermic Intraperitoneal Chemotherapy in Ovarian Cancer,” N. Engl. J. Med., vol. 378, no. 14, 2018. 10.1056/NEJMc1802033 29619814

[b0010] Aletti G.D. (2007). A new frontier for quality of care in gynecologic oncology surgery: Multi-institutional assessment of short-term outcomes for ovarian cancer using a risk-adjusted model. Gynecol. Oncol..

[b0015] Aletti G.D., Dowdy S.C., Podratz K.C., Cliby W.A. (2007). Relationship among surgical complexity, short-term morbidity, and overall survival in primary surgery for advanced ovarian cancer. Am. J. Obstet. Gynecol..

[b0020] Antonio C.C.P. (2022). Cytoreductive Surgery With or Without HIPEC After Neoadjuvant Chemotherapy in Ovarian Cancer: A Phase 3 Clinical Trial. Ann. Surg. Oncol..

[b0025] Bisch S.P. (2021). Outcomes of enhanced recovery after surgery (ERAS) in gynecologic oncology – A systematic review and meta-analysis. Gynecol. Oncol..

[b0030] Cooksley T.J., Haji-Michael P. (2011). Post-operative critical care management of patients undergoing cytoreductive surgery and heated intraperitoneal chemotherapy (HIPEC). World J. Surg. Oncol..

[b0035] Elias K.M. (2019). The Reporting on ERAS Compliance, Outcomes, and Elements Research (RECOvER) Checklist: A Joint Statement by the ERAS® and ERAS® USA Societies. World J. Surg..

[b0040] Hübner M. (2020). Guidelines for Perioperative Care in Cytoreductive Surgery (CRS) with or without hyperthermic IntraPEritoneal chemotherapy (HIPEC): Enhanced Recovery After Surgery (ERAS®) Society Recommendations — Part II: Postoperative management and special considerations. Eur. J. Surg. Oncol..

[b0045] Hübner M. (2020). Guidelines for Perioperative Care in Cytoreductive Surgery (CRS) with or without hyperthermic IntraPEritoneal chemotherapy (HIPEC): Enhanced recovery after surgery (ERAS®) Society Recommendations — Part I: Preoperative and intraoperative management. Eur. J. Surg. Oncol..

[b0050] Kehlet H. (1997). Multimodal approach to control postoperative pathophysiology and rehabilitation. Br. J. Anaesth..

[b0055] Ledermann J.A. (2024). ESGO–ESMO–ESP consensus conference recommendations on ovarian cancer: pathology and molecular biology and early, advanced and recurrent disease. Ann. Oncol..

[b0060] C. Liakou et al., “Interval Cytoreductive Surgery and Heated Intraperitoneal Chemotherapy for Ovarian Cancer: Report of a Single-center Experience,” Anticancer Res., vol. 43, no. 10, pp. 4593 LP – 4599, Oct. 2023. 10.21873/anticanres.16653 37772571

[b0065] Lim M.C. (2022). Survival After Hyperthermic Intraperitoneal Chemotherapy and Primary or Interval Cytoreductive Surgery in Ovarian Cancer: A Randomized Clinical Trial. JAMA Surg..

[b0070] Ljungqvist O., Scott M., Fearon K.C. (Mar. 2017). Enhanced Recovery After Surgery: A Review. JAMA Surg..

[b0075] Lu P.W. (2020). Cytoreductive Surgery and HIPEC in an Enhanced Recovery After Surgery Program: A Feasibility Study. J. Surg. Res..

[b0080] H. D. Mogal *et al.*, “Routine Admission to Intensive Care Unit After Cytoreductive Surgery and Heated Intraperitoneal Chemotherapy: Not Always a Requirement,” *Ann. Surg. Oncol.*, vol. 23, no. 5, 2016. 10.1245/s10434-015-4963-8 PMC4816664 26572753

[b0085] Nelson G. (2016). Guidelines for postoperative care in gynecologic/oncology surgery: Enhanced Recovery after Surgery (ERAS®) Society recommendations - Part II. Gynecol. Oncol..

[b0090] Nelson G. (2019). Guidelines for perioperative care in gynecologic/oncology: Enhanced Recovery after Surgery (ERAS) Society recommendations - 2019 update. Int. J. Gynecol. Cancer.

[b0095] Nelson G., Fotopoulou C., Taylor J. (2023). Enhanced Recovery after Surgery (ERAS®) Society guidelines for gynecologic oncology: addressing implementation challenges - 2023 update. Gynecol. Oncol..

[b0100] Robella M. (2023). Enhanced Recovery after Surgery (ERAS) Program for Patients with Peritoneal Surface Malignancies Undergoing Cytoreductive Surgery with or without HIPEC: A Systematic Review and a Meta-Analysis. Cancers.

[b0105] van Kooten J.P. (2021). Nasogastric- Vs. Percutaneous gastrostomy tube for prophylactic gastric decompression after cytoreductive surgery with hyperthermic intraperitoneal chemotherapy. Pleura and Peritoneum.

[b0110] von Elm E., Altman D.G., Egger M., Pocock S.J., Gøtzsche P.C., Vandenbroucke J.P. (2014). The strengthening the reporting of observational studies in epidemiology (STROBE) statement: Guidelines for reporting observational studies. Int. J. Surg..

[b0115] Webb C.A.J., Weyker P.D., Moitra V.K., Raker R.K. (2013). An overview of cytoreductive surgery and hyperthermic intraperitoneal chemoperfusion for the anesthesiologist. Anesth. Analg..

[b0120] Yang R. (2023). Effect of standardized fluid management on cardiac function after CRS + HIPEC in patients with PMP: a single-center case-control study. Int. J. Hyperth..

[b0125] P.Y. Zhao et al., “Clinical Efficacy and Safety of Hyperthermic Intraperitoneal Chemotherapy in Colorectal Cancer Patients at High Risk of Peritoneal Carcinomatosis: A Systematic Review and Meta-Analysis,” Frontiers in Surgery, vol. 7. 2020. 10.3389/fsurg.2020.590452 PMC7705102 33282908

